# Bridging HRSA Priority Gaps in Geriatrics Education: Insights from Geriatric Fast Facts Usage Data

**DOI:** 10.1093/geroni/igaf122.3262

**Published:** 2025-12-31

**Authors:** Edmund Duthie, Angela Beckert, Kathryn Denson, Amanda Szymkowski, Deborah Simpson, Steven Denson

**Affiliations:** Medical College of Wisconsin, Milwaukee, Wisconsin, United States; Medical College of Wisconsin, Milwaukee, Wisconsin, United States; Medical College of Wisconsin, Milwaukee, Wisconsin, United States; Medical College of Wisconsin, Milwaukee, Wisconsin, United States; Advocate Aurora Health, Milwaukee, Wisconsin, United States; Medical College of Wisconsin, Milwaukee, Wisconsin, United States

## Abstract

The Health Resources & Services Administration (HRSA) has identified 11 priority topics for interprofessional training and education to address the primary care needs of older adults. Healthcare professionals and learners increasingly rely on online resources for focused education. The Geriatric Fast Facts (GFFs) website is a globally accessed mobile enabled website for geriatrics education. Analyzing user engagement data from this site can identify gaps between expert-defined priorities and user-identified needs, informing targeted educational dissemination efforts. 107 GFFs were mapped to the 11 HRSA age/dementia-friendly priority topics. Google Analytics usage data from 2/25/2024 – 2/24/2025 was analyzed to assess GFF user engagement, including usage per topic and alignment with HRSA priorities. 42% (45/107) of GFFs were associated with 7 HRSA priorities. Of the top 25 viewed GFFs, 8 (32%) were associated with 3 HRSA priorities: Elder Justice (n = 3), Early detection, diagnosis and treatment of dementia (n = 4), and ADRD/ other mental health conditions (n = 1). Elder Justice-associated GFFs had the highest engagement (3,008 views for 7 GFFs), while Health Inequities, Disparities, and Social Determinants of Health had the lowest (35 views for one GFF). Four HRSA priorities were not associated with any GFF. User engagement with GFFs shows selective alignment with HRSA priorities with highest user interest in Elder Justice. This gap analysis reveals the need to enhance geriatrics education by expanding content related to underrepresented HRSA priorities (e.g., abuse prevention and legal protections) and the need to enhance dissemination strategies for under accessed topics.

